# The Airway As the First Signal of Relapse: Metachronous Endobronchial Metastasis From Cutaneous Melanoma

**DOI:** 10.7759/cureus.101542

**Published:** 2026-01-14

**Authors:** Ana Raquel S Afonso, Sofia Gomes Salgueira, Rafael Noya, Joelma Silva, Liliana Ribeiro, Susana Pipa, Maria João Silva, Bebiana Conde

**Affiliations:** 1 Pulmonology Department, Unidade Local de Saúde de Trás-os-Montes e Alto Douro, Vila Real, PRT; 2 Medical Oncology, Instituto Português de Oncologia do Porto Francisco Gentil, EPE, Porto, PRT

**Keywords:** bronchoscopy, case report, diagnosis of rare cases, endobronchial melanoma, endobronchial neoplasms, haemoptysis, immunotherapy, melanoma, melanoma metastasis, pembrolizumab

## Abstract

Melanoma is a malignant tumour with a recognised potential for late recurrence. Although pulmonary involvement is common in advanced disease, endobronchial metastasis is rare and may present with non-specific respiratory symptoms.

We report the case of an 85-year-old woman with a history of stage IIA melanoma of the right thumb treated with amputation in 2014, and no evidence of disease until loss to follow-up in 2022. Nearly ten years after initial treatment, she developed progressive cough, haemoptysis and dyspnoea. Chest CT revealed right lower-lobe atelectasis with post-obstructive changes. Bronchoscopy demonstrated a friable endobronchial lesion obstructing the right B9 segment. Histopathological examination showed spindle-cell malignant melanoma with diffuse Melan-A and vimentin expression and focal S100 positivity, while epithelial markers were negative. Molecular testing confirmed BRAF wild-type status, supporting metastatic recurrence.

The patient subsequently developed recurrent haemoptysis and worsening dyspnea requiring hospitalisation. Palliative radiotherapy achieved temporary haemostatic benefit relief. Pembrolizumab was initiated, but disease progression ultimately occurred after several months, accompanied by functional decline. Brain imaging performed after a seizure episode revealed multiple cerebral metastases. She transitioned to best supportive care and died in early 2025.

This case highlights a rare metachronous endobronchial recurrence of melanoma occurring a decade after primary tumour resection. Endobronchial metastasis should be considered in patients with a remote history of melanoma presenting with new respiratory symptoms. Prompt diagnosis through bronchoscopy and immunohistochemistry enables timely implementation of palliative and supportive management.

## Introduction

Melanoma is an aggressive neoplasm with high metastatic potential, largely due to the intrinsic ability of melanocytes to disseminate early through both hematogenous and lymphatic pathways [1,2]. While most of the cases originate in areas of cumulative sun damage, mucosal melanomas, arising in a surface epithelium other than skin, constitute about 1.3% of all melanomas [3]. Primary mucosal melanoma of the tracheobronchial tree is rare but should be considered in the differential diagnosis of endobronchial lesions [3].

Cutaneous melanoma metastases may occur many years after the initial diagnosis, reflecting the tumour’s well-recognised ability for prolonged dormancy and later recurrence [4,5]. When distant metastases occur, the most commonly affected sites include the liver, central nervous system, bone and lungs [6]. Pulmonary involvement typically presents as parenchymal nodules, and direct involvement of the endobronchial tree is distinctly uncommon [7-9], accounting for approximately 4.5% of endobronchial metastases arising from extra-thoracic primary tumours [7].

The histopathological distinction between the two entities, metastasis or primary mucosal melanoma, is frequently challenging, and the past medical history of previous melanoma must be taken into account, as well as a thorough physical examination, including the eye [10].

Clinical manifestations of endobronchial disease are often non-specific and may lead to airway obstruction, post-obstructive pneumonia, haemoptysis, and progressive dyspnoea [11]. Due to the rarity of this presentation, diagnosis requires a high index of suspicion and must be confirmed by bronchoscopy with histopathological and immunohistochemical evaluation [7].

Management is challenging and is frequently palliative, aiming at symptom control and maintenance of airway patency through approaches such as bronchoscopic tumour debulking or haemostatic radiotherapy. Systemic therapy is selected based on tumour molecular profile and patient characteristics, namely performance status and comorbidities. Although the prognosis of patients with melanoma has dramatically changed with the advent of immunotherapy and BRAF and MEK inhibitors, melanoma metastasis to the endobronchial tree usually carries an aggressive behaviour [9,10,12].

Given the extreme rarity of endobronchial involvement by melanoma, particularly when it occurs as a very late event after apparently curative treatment, reporting such cases remains relevant to expand the limited available evidence. This case is presented to underline the potential for late melanoma relapse, the diagnostic challenges in distinguishing metastatic disease from primary endobronchial melanoma, and the importance of maintaining clinical suspicion in patients with a remote history of melanoma who present with new respiratory symptoms.

## Case presentation

An 85-year-old woman, a non-smoker, with a past medical history of hypertension, obesity, chronic heart failure, atrial fibrillation on long-term anticoagulation, and a prior left lower limb deep venous thrombosis, had undergone treatment for breast cancer in 2009 with no documented recurrence, and total thyroidectomy in 2010 due to multinodular goitre.

In 2014, she was diagnosed with an ulcerated melanoma of the right thumb. According to the clinical history, she had undergone a local skin excision with flap reconstruction approximately four years earlier, in another country, and no additional details regarding that procedure were available. She subsequently developed a local recurrence, for which a biopsy confirmed melanoma. Definitive surgical management consisted of amputation of the distal phalanx at the level of the interphalangeal joint. Histopathological examination revealed an acral melanoma in vertical growth phase, Clark level IV, with a Breslow thickness of 1.40 mm, mitotic rate of 5/mm2, and ulcerated. There was no evidence of lymphovascular or perineural invasion, and surgical margins were negative, the closest being 3 mm from the in situ component. The final pathological staging was pT2bN0M0, Stage IIA. No adjuvant therapy was recommended, and she remained under regular oncologic surveillance until 2022, when she ceased attending scheduled follow-up appointments on her own initiative.

**Figure 1:**
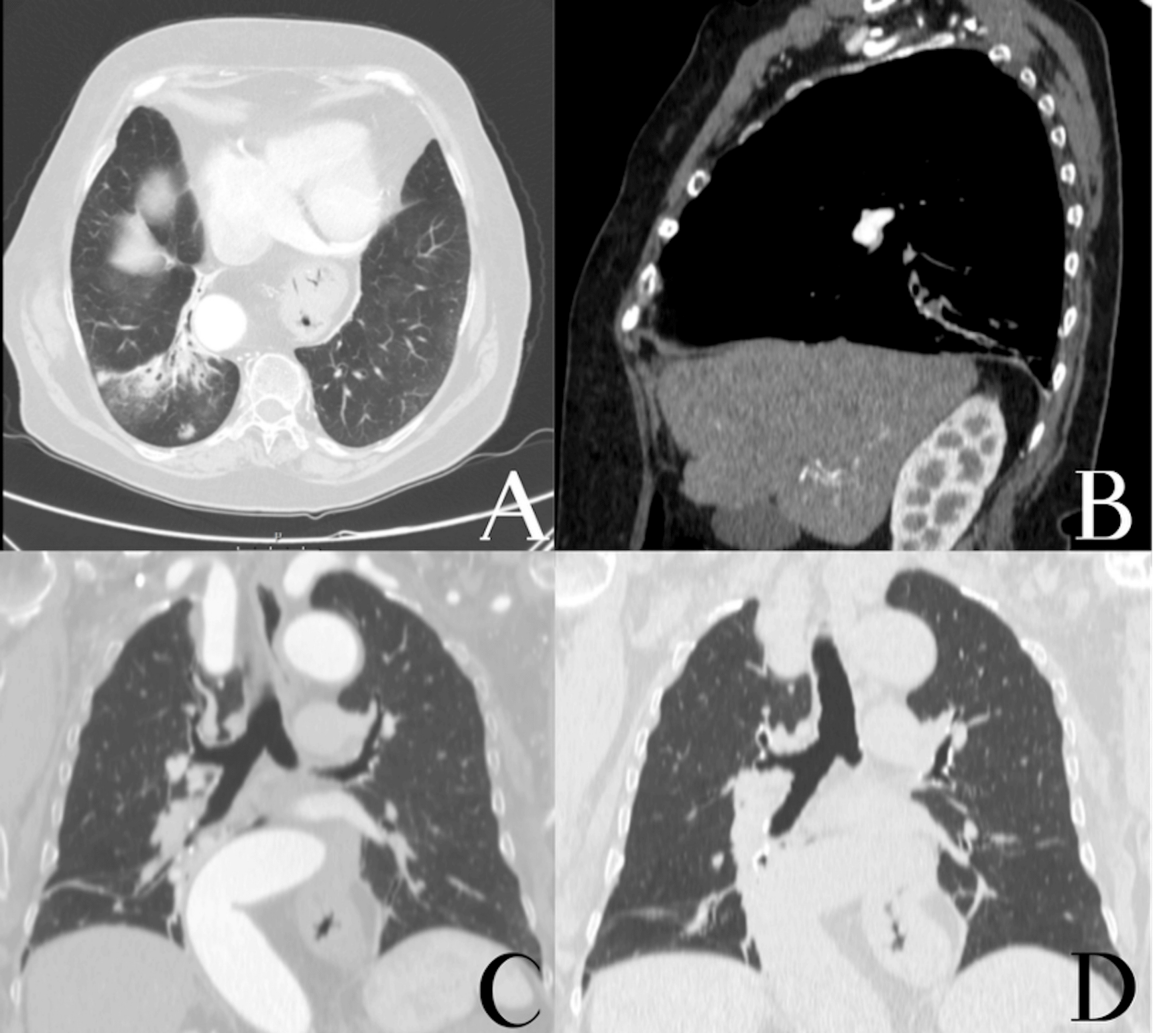
Contrast-enhanced chest computed tomography scan of the patient performed in March 2024. (A)- Axial view showing an area of increased attenuation with traction bronchiolectasis and volume loss in the posterior segment of the right lower lobe, consistent with subsegmental atelectasis and post-obstructive changes. A subpleural nodule with smooth contours and a small calcified focus, measuring approximately 16 mm, is also visible. (B)- Sagittal reconstruction demonstrating the posterior basal right lower lobe atelectatic change. (C-D)- Coronal views highlighting the extent of the atelectatic and fibro-scar alterations in the posterior right lower lobe.

Her baseline functional status was ECOG 1. In September 2023, she developed progressively worsening cough and intermittent haemoptysis, initially scant but increasing in frequency, associated with exertional dyspnoea, for which she was referred to a pulmonology appointment. A contrast-enhanced chest computed tomography (CT-scan) in March 2024 showed atelectasis of the posterior basal segment of the right lower lobe with bronchiolectasis and volume loss, consistent with post-obstructive changes, without mediastinal, hilar or axillary lymphadenopathy (Fig 1).

**Figure 2:**
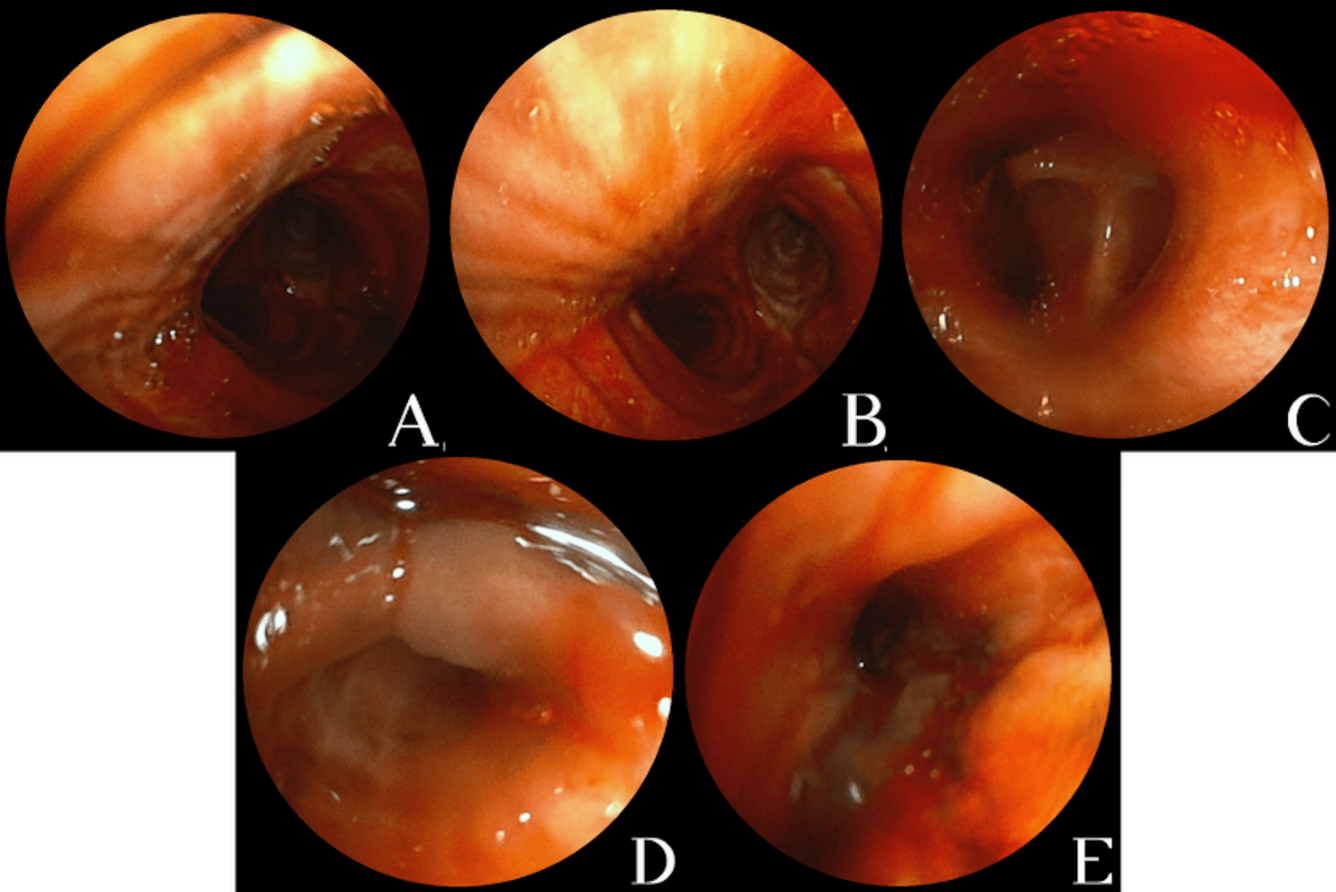
Flexible bronchoscopy performed in April 2024. (A-C)- Diffuse fresh blood throughout the tracheobronchial tree, with markedly hypervascular bronchial mucosa. (D-E)- Friable exophytic endobronchial mass in the right B9 segmental bronchus, causing partial luminal obstruction, identified as the likely source of bleeding; bronchial biopsies were obtained from this site.

Flexible bronchoscopy performed in April 2024 revealed diffuse fresh blood throughout the tracheobronchial tree, clots in segmental bronchi of the right lower lobe, diffusely hypervascular mucosa, and a friable exophytic endobronchial lesion in the right B9 segment causing partial luminal obstruction (Fig 2).

**Figure 3:**
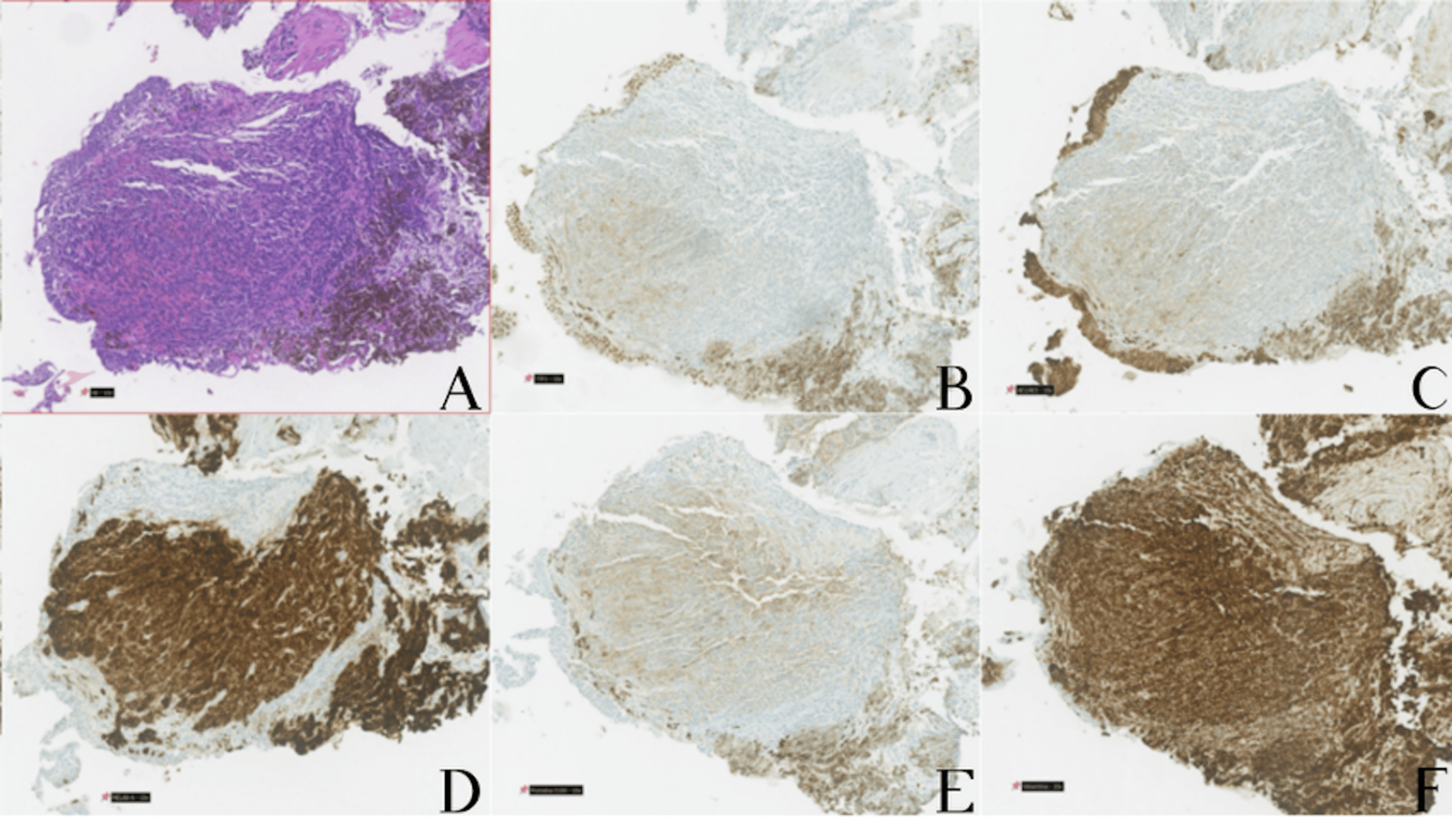
Pathological and immunohistochemical findings from the bronchoscopy biopsy specimen, x10. (A)- Haematoxylin and eosin (H&E) stain showing spindle-cell malignant melanoma infiltrating bronchial mucosa. (B)- TTF-1 immunostaining, negative in the tumor. (C)- Lack of pancytokeratin (AE1/AE3) staining in the neoplastic cells (with positive control in entrapped bronchial epithelium). (D)- Diffuse Melan-A expression in the neoplastic population. (E)- Focal S100 protein expression in the tumor cells. (F)- Diffuse vimentin expression in the spindle-cell melanoma.

Histological examination of the bronchial biopsies showed fragments of bronchial mucosa infiltrated by malignant melanoma composed of spindle-cell neoplastic elements. Immunohistochemistry demonstrated diffuse expression of vimentin and Melan-A in the neoplastic cell population, focal expression of S100, and absence of staining for TTF-1, pancytokeratins (AE1/AE3), or CD34, the latter two being positive only in entrapped bronchial epithelium (Fig 3). This immunophenotypic profile confirmed a melanocytic neoplasm and excluded a primary pulmonary carcinoma. In the absence of evidence of another primary malignancy, and given the patient’s history of cutaneous melanoma, these findings were consistent with a metachronous endobronchial recurrence of the previously diagnosed melanoma, resulting in right lower lobe obstructive atelectasis. BRAF mutation testing was negative.

Haemoptysis and dyspnoea worsened, leading to hospital admission. Anticoagulation was permanently discontinued due to malignant haemoptysis and high bleeding risk. Aminocaproic acid and codeine were initiated, resulting in gradual improvement. Haemoglobin reached a nadir of 10.9 g/dL, recovering without transfusion.

A thoracoabdominopelvic CT scan in May 2024 showed persistent right lower lobe collapse and a 20 mm pulmonary nodule in the same lobe, along with three small subcapsular hypervascular hepatic nodules (segments II and IV), suspicious for metastases. Brain MRI at this time excluded intracranial metastases.

By June 2024, she required continuous domiciliary long-term oxygen therapy at 3 L/min due to persistent dyspnoea. In July 2024, she was re-admitted with recurrent haemoptysis and respiratory distress and underwent palliative haemostatic radiotherapy, receiving 8 Gy in a single fraction (3DCRT photon technique) to the right hilar region. A CT scan demonstrated persistent right lower lobe consolidation with increased opacification of the bronchial lumen, consistent with progression of the endobronchial lesion, and small mediastinal lymph nodes up to 13 x 9 mm, without new thoracic metastatic lesions. Given BRAF wild-type disease, palliative systemic treatment with the anti-PD1 monoclonal antibody pembrolizumab (200 mg every 3 weeks) was initiated on July 11, 2024. Following radiotherapy and initiation of pembrolizumab, there was an initial reduction in haemoptysis and dyspnoea improvement, with transient symptomatic stabilization. Response evaluation with CT-scan revealed stable disease as the best response.

At oncology appointment in January 2025, however, she exhibited progressive symptomatic decline, with dyspnoea on minimal exertion, intermittent haemoptysis, and marked fatigue, corresponding to ECOG performance status 3. Examination revealed reduced breath sounds on the right hemithorax and bilateral symmetrical lower-limb oedema. Laboratory studies showed grade 1 anaemia (Hb 10 g/dL) with iron deficiency (transferrin saturation 6%). Clinical progression was assumed, pembrolizumab was discontinued six months after initiation, and she was referred to palliative care for symptomatic management.

In February 2025, she experienced an episode of haemoptysis followed by a generalized tonic-clonic seizure. Brain CT revealed multiple cerebellar and supratentorial metastatic lesions without hydrocephalus. She was admitted for comfort-focused care, seizure prophylaxis and management of cerebral oedema. She died during hospitalization in the context of disease progression.

## Discussion

This case illustrates a rare metachronous endobronchial recurrence of melanoma occurring a decade after primary tumour resection. Although pulmonary metastases are relatively common in advanced melanoma, direct involvement of the endobronchial tree is distinctly uncommon, representing only a small proportion of secondary airway tumours [9]. In published series of endobronchial metastases, melanoma accounts for a minority of primary sites, with an estimated prevalence of approximately 4.5% among endobronchial metastatic locations attributed to primary tumours, highlighting the rarity of this presentation and explaining why available evidence is largely limited to small case series and individual reports [7,12].

Late endobronchial recurrence, particularly beyond 10 years after initial diagnosis, is exceptionally rare, with only isolated case reports described in the literature [8,13,14]. Overall, the reported incidence of late melanoma recurrence ranges between 0.98% and 6.7% [15,16]. The interval between primary melanoma diagnosis and development of endobronchial metastasis varies widely. In the largest series focusing on endobronchial metastases from melanoma, the median time from primary diagnosis to airway involvement was approximately 48 months (range 0-120 months) [12]. Other studies suggest that melanoma may behave differently from other primary tumours with respect to airway involvement, with some reports describing substantially longer latency periods for endobronchial metastasis [6,7,12]. This variability appears to be influenced by the histology of the primary tumour and by the presence of synchronous versus metachronous metastatic disease [12].

In our report, symptomatic endobronchial recurrence occurred around 10 years after initial resection, supporting the concept of late melanoma recurrence. Very late recurrences, including cases occurring several decades after initial treatment, have been described, although such events remain exceptional [8,9,14]. Factors that have been associated with a higher risk of late melanoma recurrence include tumour thickness greater than 2 mm, younger age at diagnosis (< 40 years), and Clark level IV or V [17].

The presence of concurrent metastatic disease is another point of comparison. In the largest published melanoma series, approximately 79% of patients had metastases at other sites at the time endobronchial disease was diagnosed, and overall prognosis was poor, with a reported median survival of around six months (range 1-46 months) [12]. These findings suggest that endobronchial involvement most often reflects advanced systemic dissemination rather than isolated airway disease. When endobronchial disease occurs as the dominant or isolated site of recurrence, outcomes may be comparatively better; however, robust comparative evidence is lacking, given the rarity of this presentation and the small sample studies [12]. Prognosis also appears to be strongly related to the site of recurrence [18,19].

Clinically, the patient’s presentation with progressive cough, intermittent haemoptysis, and exertional dyspnoea is characteristic of endobronchial metastases but remains non-specific. As reported in prior studies, clinical and radiologic findings are often indistinguishable from primary bronchogenic carcinoma and may closely overlap with primary lung malignancies [20,21]. This reinforces the central role of bronchoscopy as the decisive investigation, allowing direct visualisation of the lesion, assessment of airway compromise, and tissue sampling for definitive diagnosis. Histopathology with immunohistochemistry remains essential to distinguish metastatic melanoma from epithelial tumours. The demonstration of melanoma markers such as S100 and Melan-A, together with the absence of epithelial markers, provides strong confirmatory evidence and avoids misclassification as primary lung cancer, which would have significant therapeutic implications.

Several mechanisms have been proposed to explain the development of endobronchial metastases, including haematogenous dissemination with implantation in the bronchial mucosa, direct bronchial invasion from adjacent parenchymal lesions, and lymphatic spread. which may account for variability in endobronchial distribution and the extent of airway involvement across cases [22,23]. The exceptionally delayed recurrence observed in this case is consistent with the well-recognised phenomenon of late melanoma relapse. Late recurrence, defined as occurring 10 years or more after initial diagnosis, has been reported at low but clinically relevant rates in melanoma cohorts [18,19]. Data from early-stage cutaneous melanoma suggest that late recurrence may occur even after stage I disease and does not always correlate with classical primary tumour characteristics, supporting the hypothesis that long-term tumour dormancy and immune surveillance play an important role [19]. These observations provide a biologically plausible explanation for the prolonged disease-free interval followed by late airway recurrence observed in this patient.

Another aspect that deserves consideration in this case is the theoretical distinction between a late endobronchial metastasis from a previously treated cutaneous melanoma and the possibility of a primary endobronchial melanoma. Although exceedingly rare, primary endobronchial melanoma has been described in isolated reports [24]. However, establishing this diagnosis requires exclusion of a previous or synchronous primary melanoma at another site. In our patient, no new cutaneous, mucosal or ocular primary lesion was identified despite appropriate clinical evaluation, and there was a well-documented history of prior cutaneous melanoma. Taken together, these findings strongly support the interpretation of the endobronchial lesion as a metastatic recurrence rather than a new primary airway melanoma.

Consistent with published series, management of endobronchial melanoma metastasis is predominantly palliative and should be individualised according to performance status, extent of extra-airway metastatic disease and degree of airway obstruction [25-27]. Bronchoscopic interventions may provide meaningful symptomatic relief in cases of central airway obstruction and/or haemoptysis, including endoluminal debulking techniques. Endobronchial stent placement can be considered in selected cases, recognising that tumour progression and re-obstruction are common and that a clear survival benefit from specific endobronchial modalities has not been consistently demonstrated [12,25-27].

Systemic therapy, guided by BRAF mutation and performance status, may provide temporary disease stabilisation, although outcomes remain strongly influenced by overall metastatic burden, in keeping with the poor median survival reported in published series of endobronchial melanoma metastases [12]. In selected patients, surgical resection may be considered. However, recurrence rates remain high and are often not feasible due to disseminated disease [28].

By contrast, in our case, therapeutic options were further limited by patient- and tumour-related factors. The tumour was BRAF-wild type, precluding the use of targeted therapy, and although recent advances in immunotherapy have significantly improved outcomes in metastatic melanoma, their benefit must be weighed against performance status, comorbidities and overall disease burden. At the time of diagnosis, the patient had advanced metastatic disease and was elderly and functionally compromised, factors that limited the feasibility of aggressive systemic treatment. Consequently, management focused primarily on symptom control. Endobronchial treatment strategies were considered strictly palliative, and haemostatic radiotherapy was performed, resulting in temporary improvement of haemoptysis and respiratory symptoms. This clinical course is consistent with published series, in which endobronchial interventions provide short-term symptomatic relief but do not alter the overall poor prognosis associated with advanced metastatic melanoma [12,25-27].

Overall, we intend to reinforce several important considerations. Late recurrence is a well-recognised but under-appreciated feature of melanoma, and rigid follow-up cut-offs may miss a small but clinically significant subset of patients who relapse late [13,18,19]. Although rare, endobronchial metastasis should remain in the differential diagnosis of haemoptysis or new respiratory symptoms in patients with a remote history of melanoma, as prompt bronchoscopy and accurate histological classification directly inform appropriate interventions [7,12]. The rarity of endobronchial metastasis explains the limited number of available studies and the small cohorts on which current evidence is based.

## Conclusions

The case presented reinforces the importance of recognising new-onset respiratory symptoms in patients with a history of malignant melanoma, as well as the crucial role of bronchoscopy in the diagnosis of this rare presentation of metastatic melanoma. Despite the unfavourable prognosis, early identification of endobronchial metastatic disease may enable prompt implementation of targeted therapeutic interventions or appropriate palliative management aimed at optimising symptom control and enhancing quality of life.
